# Heavy Metal Detection in *Fritillaria thunbergii* Using Laser-Induced Breakdown Spectroscopy Coupled with Variable Selection Algorithm and Chemometrics

**DOI:** 10.3390/foods12061125

**Published:** 2023-03-07

**Authors:** Muhammad Hilal Kabir, Mahamed Lamine Guindo, Rongqin Chen, Xinmeng Luo, Wenwen Kong, Fei Liu

**Affiliations:** 1 College of Biosystems Engineering and Food Science, Zhejiang University, 866 Yuhangtang Road, Hangzhou 310058, China; 2 Department of Agricultural and Bio-Resource Engineering, Abubakar Tafawa Balewa University, Bauchi PMB 0248, Nigeria; 3 College of Mathematics and Computer Science, Zhejiang A&F University, Hangzhou 311300, China; 4 Key Laboratory of Spectroscopy Sensing, Ministry of Agriculture and Rural Affairs, Hangzhou 310058, China

**Keywords:** laser-induced breakdown spectroscopy, *Fritillaria thunbergii*, heavy metals, chemometrics, variable selection, machine learning

## Abstract

Environmental and health risks associated with heavy metal pollution are serious. Human health can be adversely affected by the smallest amount of heavy metals. Modeling spectrum requires the careful selection of variables. Hence, simple variables that have a low level of interference and a high degree of precision are required for fast analysis and online detection. This study used laser-induced breakdown spectroscopy coupled with variable selection and chemometrics to simultaneously analyze heavy metals (Cd, Cu and Pb) in *Fritillaria thunbergii*. A total of three machine learning algorithms were utilized, including a gradient boosting machine (GBM), partial least squares regression (PLSR) and support vector regression (SVR). Three promising wavelength selection methods were evaluated for comparison, namely, a competitive adaptive reweighted sampling method (CARS), a random frog method (RF), and an uninformative variable elimination method (UVE). Compared to full wavelengths, the selected wavelengths produced excellent results. Overall, RC^2^, RV^2^, RP^2^, RSMEC, RSMEV and RSMEP for the selected variables are as follows: 0.9967, 0.8899, 0.9403, 1.9853 mg kg^−1^, 11.3934 mg kg^−1^, 8.5354 mg kg^−1^; 0.9933, 0.9316, 0.9665, 5.9332 mg kg^−1^, 18.3779 mg kg^−1^, 11.9356 mg kg^−1^; 0.9992, 0.9736, 0.9686, 1.6707 mg kg^−1^, 10.2323 mg kg^−1^, 10.1224 mg kg^−1^ were obtained for Cd Cu and Pb, respectively. Experimental results showed that all three methods could perform variable selection effectively, with GBM-UVE for Cd, SVR-RF for Pb, and GBM-CARS for Cu providing the best results. The results of the study suggest that LIBS coupled with wavelength selection can be used to detect heavy metals rapidly and accurately in Fritillaria by extracting only a few variables that contain useful information and eliminating non-informative variables.

## 1. Introduction

The World Health Organization (WHO) reports that herbal medicines remain the primary treatment for a number of diseases in developing countries [1]. The use of nutraceuticals and medicinal products derived from medicinal herbs is increasing even in developed countries [1]. The consumption of healthy herbs is currently receiving considerable attention, and there is a focus on consuming products that are as natural as possible and have as little contamination as possible. The demand for nutraceuticals and herbal dietary supplements has increased significantly in recent years. Plants and other natural materials are used to make these products. There is, therefore, a high probability of heavy metal contamination [2]. The food chain may introduce heavy metals to humans through the accumulation of heavy metals in the environment [3,4,5,6]. There are numerous anthropogenic sources and activities that lead to heavy metal contamination of the environment, such as mining, traffic, agriculture, and industrial processes. However, heavy metals are naturally present in the Earth’s crust [6,7]. Risks associated with heavy metals can be represented in a variety of ways [8]. They can be found in the air we breathe, the food we eat, and the water we drink. They can also be found in soil and dust, which can be inhaled or ingested. As a result, they can enter the body and cause health problems.

In traditional Chinese medicine (TCM), heavy metals such as arsenic (As), mercury (Hg), lead (Pb), copper (Cu) and cadmium (Cd) are of particular concern [2]. Human health is at risk from these pollutants. The intake of excessive amounts of heavy metals is detrimental to the human body because it results in neurotoxicity, organic damage, and diseases of the skin and blood [9,10].

*Fritillaria thunbergii* Miq is a perennial herbaceous plant that is native to the provinces of Zhejiang, Jiangsu, and Anhui in China [11]. A dry bulb of Thunbergii fritillaria (Zhebeimu), a plant from the family of Thunbergii, is frequently used in Chinese medical clinical practice for treating coughs caused by wind and phlegm heats, as well as bronchitis, inflammation, hypertension, gastric ulcer, diarrhea, and bacterial infections [12]. Furthermore, Zhebeimu is extensively used to treat leukemia that is resistant to drugs [13]. Heavy metals have become one of the most serious safety concerns due to increasingly stringent TCM regulations [14,15,16]. Thus, accurate detection of heavy metal concentrations in TCM is crucial. Among the methods commonly used for heavy metal detection are atomic absorption spectroscopy (AAS), atomic fluorescence spectroscopy (AFS), X-ray fluorescence spectroscopy (XRFS), inductively coupled plasma optical emission spectroscopy (ICP-OES) [17,18], and electrochemical methods, especially stripping and cyclic voltammetry, which are commonly used methods for detection. Voltammetry is a sensitive electrochemical method that is widely used for heavy metal detection [19,20,21]. A traditional heavy metal detection method involves sampling, pretreatment, and laboratory chemical analysis; all of which are time-consuming, costly, and require extensive preparation [22,23].

Multi-elemental detection can be achieved through laser-induced breakdown spectroscopy (LIBS) [24]. LIBS uses pulsed laser ablation to create plasma on a sample and then detects and analyzes the emission light emanating from the plasma. With LIBS, a sample does not need to be prepared prior to analysis, thus allowing for rapid results [25], minimum requirements for small samples [26], and cost-effective instrumentation [27]. LIBS is widely used in a wide variety of industries [28], including mining [29], plastics [30], biomedicine [31,32], food [33], and the environment [34]. LIBS can provide information on the composition of samples within a short period of time, as well as the element content of samples. In comparison with other detection technologies, LIBS has many advantages, such as the requirement for fewer samples, the lack of complex pretreatment, the ability to measure multiple elements simultaneously, and the possibility of rapid implementation [35].

A number of studies have been conducted in recent years that focus on using LIBS to detect heavy metal pollution. Wang et al. [36] applied LIBS to detect cadmium content in rice. The LIBS analysis of rice stems demonstrated that it is an effective method for detecting cadmium. Su et al. [37] simultaneously and quantitatively analyzed the heavy metals in *Sargassum fusiforme* by using laser-induced breakdown spectroscopy. Liu et al. (2020) [38] analyzed cosmetics for trace lead and cadmium through laser-induced breakdown spectroscopy and ultrasound-assisted extraction. Rehan et al. [39] analyzed henna paste, and fresh leaves and soils were tested with LIBS to detect lead and nutrients. Wang et al. [40] conducted an analysis of lead and copper in Ligusticum wallichii using LIBS. Lead (Pb) levels in soil were quantitatively analyzed by Zhao et al. [41]. It was demonstrated that the dual-pulse laser-induced breakdown spectroscopy (DP-LIBS) was an efficient spectroscopic tool for improving the quantitative analysis of Pb heavy metal in soil. Zhu et al. [42] performed an analysis of the content of arsenic in traditional Chinese medicine using laser-induced breakdown spectroscopy (LIBS). Rehan et al. [43] used LIBS to assess the amount of toxic heavy metals (Pb, Cr, Ni) present in different brands of face foundation powder. Zhu et al. [44] detected lead in rhododendron leaves using laser-induced breakdown spectroscopy assisted by laser-induced fluorescence. Peng et al. [45] analyzed rice leaves using collinear DP-LIBS to determine their chromium content. Yang et al. [46] determined the content of lead and cadmium in rice using LIBS. These studies are indicative of the increasing consolidation of LIBS associated with chemometrics methods for the analysis of heavy metals.

However, despite the increase of fast and clean methods for TCM analysis [47], the application of gradient-boosting machine learning algorithms has rarely been used for heavy metal prediction in TCM. Thus, detecting heavy metals in different varieties of Fritillaria based on LIBS technology combined with a gradient boosting machine is unique and important for monitoring human exposure and establishing effective environmental control strategies.

Considering the potential risks associated with heavy metals in traditional Chinese medicine (TCM), the present work investigated the feasibility of LIBS combined with chemometrics in measuring cadmium (Cd), copper (Cu) and lead (Pb) simultaneously in twelve (12) different varieties of *Fritillaria thunbergii*. However, the specific objectives were as follows: (a) to test the feasibility of the gradient boosting machine (GBM) as a method of measuring heavy metals in different varieties of Fritillaria using both full and extracted variables, (b) to verify the effectiveness of using three variable selection methods, namely, competitive adaptive reweighed sampling (CARS), random frog (RF) and uninformative variable elimination (UVE) by comparing model (GBM, SVR and PLSR) performances, and (c) to establish a quantitative analysis model for heavy metals based on full and extracted variables.

## 2. Materials and Methods

### 2.1. Sample Collection and Preparation

A total of twelve (12) different varieties of Fritillaria thunbergia were used in the experiment. The varieties were provided by the Faculty of Biosystems Engineering and Food Science (Zhejiang University, Hangzhou, China). Different copper (Cu), cadmium (Cd) and lead (Pb) samples were prepared in the laboratory using Cu(NO_3_)_2_, Cd(NO_3_)_2_. 4H_2_O and Pb (NO_3_)_2_ [40,44,46,48], respectively. Fritillaria varieties were randomly divided into eight groups to obtain samples with varying levels of Cu, Cd, and Pb. The first group was designated as a control group, whereas the remaining seven groups were designated as treatment groups. In order to accurately quantify Cu, Cd, and Pb concentrations in Fritillaria samples, the solution was artificially contaminated for 48 h at 4 °C and rinsed three times with super-pure water. This was done to simulate the effects of heavy metal pollution in the environment and to determine the best course of action for mitigating the environmental damage caused by these pollutants. Following drying at room temperature, all samples were milled at high speeds using a high-speed pulverizer (FW100, TAISITE, Tianjin, China). In order to produce pellets from these ground samples, they were compressed using a tablet compressor (FY-24, SCJS, Tianjin, China), of 1.5 cm in diameter, at a pressure of 25 kN for the duration of one minute. In total, 288 pellets were prepared.

### 2.2. Experimental Setup

**Figure 1:**
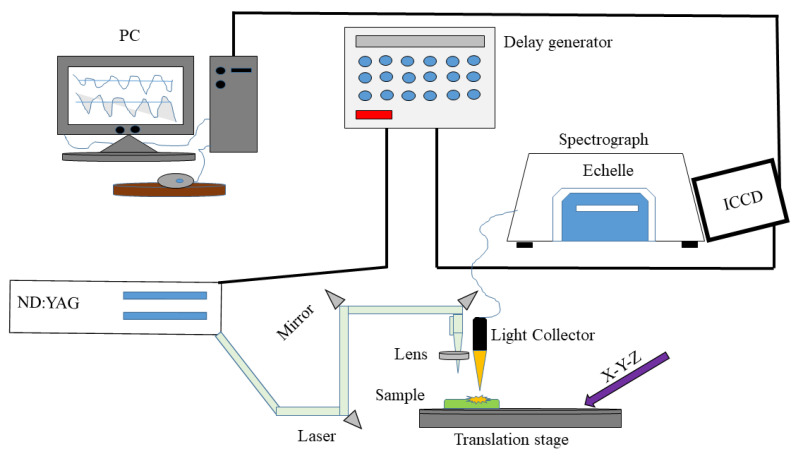
An illustration of the LIBS experimental setup.

A LIBS self-assembled schematic diagram is available in [49]; Figure 1 illustrates the methods used in this experiment. Laser pulses were generated at 532 nm with a maximum energy of 200 mJ and a pulse width of 8 ns using a Q-switched Nd: YAG pulse laser (Vlite 200, Beamtech, Beijing, China). A planoconvex lens (f = 100 mm) finally focused the laser onto the sample surface after passing through the optical system. The laser ablation generated plasma which emitted electromagnetic waves that diffused outward. In order to measure the waves, a light collector was used, and the waves were captured by a spectrometer (SR-500i-A-R, Andor Technology, Belfast, UK) along with an intensified charge-coupled device (ICCD) camera (DH334T-18F-03, Andor Technology, Belfast, UK). The laser Q-switch and ICCD camera were delayed using a delay generator (DG645, Stanford Research Systems, Sunnyvale, CA, USA). Several parameters were optimized before the experiment, including the laser energy of 60 mJ, the delay time of 1.5 µs, and the gate width of 10µs. The Fritillaria pellets were automatically placed, and the laser ablation path was controlled using a 4 × 4 array of craters designed using automatic x-y-z translation. Laser pulses accumulated five times faster in each crater. For each sample, an average of 80 spectra (4 × 4 × 5) were taken in order to reduce fluctuation between the laser points. Approximately one minute was required to collect LIBS information for one sample.

### 2.3. Determination of Heavy-Metals Reference Value

Inductively coupled plasma mass spectrometry (ICP-MS) was used to determine the contents of three (3) heavy metals (Cu, Cd, and Pb) in *Fritillaria thunbergii*. Table 1 lists the statistics of heavy metal contents in different samples. A number of procedures are involved in the ICP-MS analysis of the samples, including digestion of the sample, filtration and purification of the digestion solution, and detection of the digestion solution using ICP-MS (ELAN DRC-e, Perkin Elmer, USA). The pH value as well as all steps in the ICP-MS analysis were carried out by experimental technicians at the Zhejiang College of Biosystems Engineering and Food Science, Zhejiang University. This was a preliminary attempt to detect multiple heavy metals (Cd, Cu and Pb) in Fritillaria thubergii using the LIBS technique. The pH value was determined by measuring the electrical conductivity of the sample solution with a pH meter. The ICP-MS analysis was performed using a quadrupole-based ICP-MS system which allowed the technicians to accurately measure the concentrations of elements in the sample solution. Similar steps are described by Su et al. [37]. Unscrambler X, version 10.1 (CAMO Software AS, Oslo, Norway, 2011) was used for the descriptive statistics (file imported in MATLAB format).

### 2.4. Data Analysis

#### 2.4.1. Spectral Modeling

**Figure 2:**
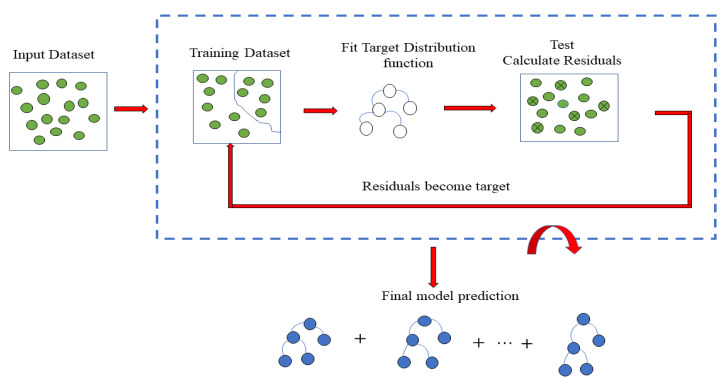
Gradient Boosting Machine Flow Chart.

The gradient boosting technique (GB) is a machine-learning method used in regression and classification problems. As a result, a prediction model is produced as an ensemble of weak prediction models. Every step evaluates the model values at each training data point, using the residuals of previous steps to minimize the loss function [50]. A GBM utilizes the best practices to avoid overfitting the classification machine. A subsample of the training data is randomly selected (without replacement) from the full dataset for each iteration in order to fit the base learner for that iteration. Figure 2 illustrates the main processes involved in gradient boosting through a simplified flow chart.

In comparison to other machine learning methods, gradient boosting machines (GBMs) have several advantages. GBM, in addition to its complex classification capability, allows soft classification, which entails calculating the probability of each sample being a member of each class, rather than labeling every sample as part of a single category (hard classification). It facilitates the assessment of the reliability of the statistical model (the potential for overfitting) and the study of the chemical-physical properties of the model, thus fostering the development of further qualitative and quantitative research studies.

In addition, it provides a natural measure of how significant each spectral feature is for classifying data, something that is usually lost in the black-box nature of many machine learning algorithms, which include artificial neural networks. The relative importance of variables is computed in GBM. A refinement of the split criterion is computed at each split in each tree (MSE for regression). Averaging the improvements made by each variable over every tree that uses that variable follows. In the split criterion, the variables with the greatest average decrease are listed as the most significant [50].When it comes to GBM modeling, there are a variety of tuning parameters available. The following variables were used in this study: (boosting_type = ‘gbdt’, num_leaves = 31, max_depth = −1, learning_rate = 0.1, n_estimators = 100). In order to forecast discrete values, SVR is an approach that uses supervised learning. By comparison, it aims to determine the hyperplane with the most points, or the line of best fit [51,52]. PLS uses projections to build linear regression models using variables and observables [53,54,55]. A significant amount of collinearity can be analyzed by this algorithm, unlike previous algorithms.

#### 2.4.2. Variable Selection Methods

Generally, LIBS data display high covariance due to the capability of the technique to measure multiple emission lines associated with the same element or species. Moreover, each peak is a result of a combination of many factors. It is therefore possible to reduce the covariance and complexity of the model by selecting variables. Selection of more explanatory variables improves the understanding of the multivariate system [56]. The removal of noisy areas, such as the extremes of each spectrometer and variables without analytical information, is also likely to increase the explained variance and enhance the accuracy of the model [56]. To select the optimal variable, variables should be selected and eliminated. In this study, three methods of variable selection were used to simplify the calculation process and improve model performance (CARS, RF, and UVE).

In CARS, wavelengths are selected using a survival-of-the-fittest principle [57]. Firstly, wavelengths with small regression coefficients are removed by using an exponentially decreasing function (EDF). An EDF equation is then used to calculate the wavelength ratio. The following steps are involved in each sampling run: (a) the Monte Carlo (MC) principle is used to sample models; (b) EDF-based wavelength selection is performed; (c) adaptive reweighted sampling is used for competitive wavelength selection and (d) cross-validation evaluation of the subset is performed [57]. A subset of wavelengths with the lowest root mean squared error of cross-validation (RMSECV) is retained as the effective wavelength, and wavelengths with little or no effective information are eliminated [58].

The RF method is iterative in nature. There are primarily three steps in the random frog algorithm: (1) A random selection of features is used to create an initialized feature subset. (2) Iteration is performed until a candidate feature subset is selected. This is accepted with a certain probability, then replaced, and this step is looped until the desired number of iterations is achieved. (3) As a measure of feature importance, the selection probability of each feature is calculated [59]. The total number of feature subsets can be determined after multiple iterations. Its selection probability can be calculated as

(1) $$P_{j}=\frac{N_{j}}{N},\;j=1,\;2,\;\ldots \ldots n$$

N_j_ denotes the frequency of the j_th_ feature, j = 1, 2, …, n, selected from the features. For each feature, N_j_ is the feature subsets after iteration, and N is the feature frequency.

Accordingly, the more optimal a feature, the more likely it is to be selected for inclusion in these subsets of features. As a result, features can be selected according to feature importance. The UVE method uses regression coefficients from a PLS model to select variables. This method is useful in eliminating non-informative variables, and the remaining variables can be used to analyze and classify chemicals [60,61].

#### 2.4.3. Model Evaluation and Calculation

In order to assess the performance of the calibration model, several evaluation indices were calculated. The point-to-point fluctuations in the spectra were reduced by area normalization [45]. The data were all normalized prior to modeling. The accuracy of the model was assessed by determining the root mean square error of calibration (RMSEC), the root mean square error of validation (RMSEV), the root mean square error of prediction (RMSEP) and the coefficient of calibration (RC^2^), coefficient of validation (RV^2^) and coefficient of prediction (RP^2^) based on the predicted results. In summary, a good calibration model should have a small RMSEC, and RMSEP, as well as large values for (RC^2^) and (RP^2^) [37,48,62,63]. The calculations were performed using Python with Scikit-Learn and the figures were generated using Origin 2022.

## 3. Results and Discussion

### 3.1. Spectra Analysis

**Figure 3:**
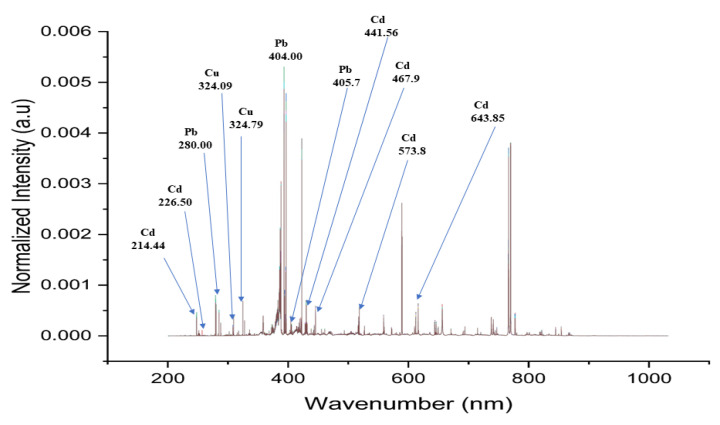
Normalized spectra of 12 Fritillaria varieties.

In LIBS, atoms and ions are expelled from a generated plasma as a result of their excited state [64]. Figure 3 illustrates the normalized spectra of 12 Fritillaria varieties. In accordance with the National Institute of Standards and Technology, USA, Electronic Database, characteristic lines for Cd, Cu, and Pb were identified. In this study, however, the purpose is to quantitatively analyze the content of three heavy metals (Cd, Cu and Pb), and because the LIBS spectra of the different varieties have similar curves, it is difficult to observe the LIBS spectra simultaneously and quantitatively to analyze the heavy metal content of Fritillaria simultaneously and quantitatively. Hence, the LIBS data must be further analyzed using chemometric methods.

### 3.2. Heavy Metals Prediction Using Full and Selected Variables

We performed LIBS analyses in accordance with best practices [65]. The full spectrum was divided into three parts: calibration (60%), validation (28%), and prediction (12%). A summary of the GBM results for the full wavelength and the selected wavelength is presented in Table 1 and Table 2. The peaks in full LIBS data usually exhibit high covariance due to the capability of measuring multiple emission lines of the same element or species and the fact that a number of x variables are required to generate a single peak. A useful range of wavelengths should be selected from the entire range of wavelengths in order to simplify the LIBS calibration models and improve prediction accuracy. As a result, three methods of variable selection (CARS, RF, and UVE) were used to select the informative variables from the whole wavelength range to simplify the calculation process and to improve the performance of the model.

#### 3.2.1. Cd Content Prediction Using Full and Selected Variables

**Figure 4:**
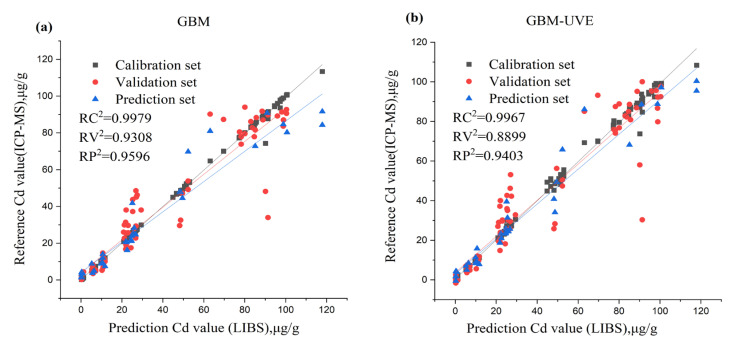
(**a**) Scatter plots of the full variables and reference value (**b**) Scatter plots of GBM combined with UVE feature selection method and reference value for the content of Cd.

All three variable selection methods (CARS, RF and UVE) were computed with SVR, PLSR and GBM to predict Cd (Table 2), and the best result was achieved with UVE-GBM. The calibration RC^2^ achieved by the model was 0.9967, the RMSEC was 1.9853 mg kg^−1^, the validation set RV^2^ achieved 0.8899, the RMSEV was 11.3934 mg kg^−1^, the prediction RP^2^ achieved 0.9403 and the RMSEP was 8.5344 mg kg^−1^. Figure 4b displays the scatter plots of the reference value and the prediction value for the Cd using the UVE-GBM.

RF-SVR and RF-PLSR also produced comparatively good results. For the RF-SVR calibration set, RC^2^ = 0.9999, RMSEC = 0.1000 mg kg^−1^, the validation set RV^2^ achieved 0.9287, the RMSEV was 9.1671 mg kg^−1^ and the prediction set was RP^2^ = 0.9322, RMSEP = 9.0933 mg kg^−1^. For the RF-PLSR calibration set RC^2^ = 0.9825, RMSEC = 4.6313 mg kg^−1^, the validation set RV^2^ achieved 0.9313, the RMSEV was 9.0014 mg kg^−1^ and the prediction set was RP^2^ = 0.9182, RMSEP = 9.9903 mg kg^−1.^ On the other hand, CARS-SVR, UVE-SVR and CARS-GBM, RF-GBM produced lower prediction outcomes (Table 3). Meanwhile, for the full spectra, GBM was the best (Table 2); for the calibration set, RC^2^ was 0.9984, RMSEC = 1.3693 mg kg^−1^, the validation set RV^2^ achieved 0.8924, the RMSEV was 11.2625 mgkg^−1^, and the prediction set was RP^2^ = 0.9139, RMSEP = 10.2494 mg kg^−1^, as shown in Figure 4a,b below. GBM combined with UVE offers superior predictions than full wavelength GBM alone. GBM-UVE performs better with Cd because it is able to handle the large number of variables and select the most important ones.

#### 3.2.2. Cu Content Prediction Using Full and Selected Variables

**Figure 5:**
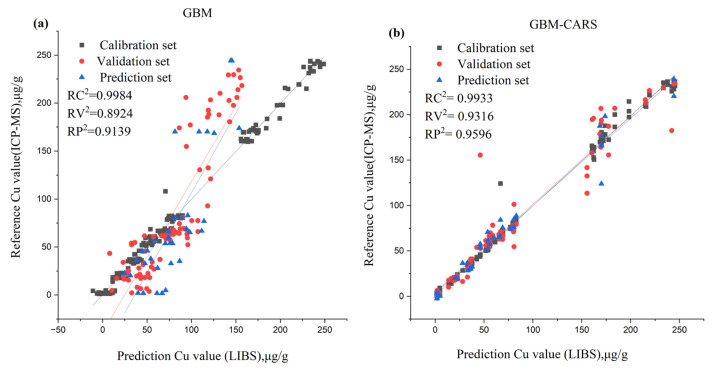
(**a**) Scatter plots of the full variables and reference value (**b**) Scatter plots of GBM combined with CARS feature selection method and reference value for the content of Cu.

For the prediction of Cu content, GBM combined with CARS achieved the best outcome with the following calibration set: RC^2^ = 0.9933, RMSEC = 5.9332 mg kg^−1^, the validation set RV^2^ achieved 0.9316, the RMSEV was 18.3779 mg kg^−1^, and the prediction set was RP^2^ = 0.9665, RMSEP = 11.9356 mg kg^−1^. This was followed by RF-SVR: for the calibration set RC^2^ = 0.9860, RMSEC = 8.5699 mg kg^−1^, the validation set RV^2^ achieved 0.9640, the RMSEV was 13.3319 mg kg^−1^, and the prediction set was RP^2^ = 0.9658, RMSEP = 12.0658 mg kg^−1^; UVE-SVR: for the calibration set RC^2^ = 0.9715, RMSEC = 12.2395 mg kg^−1^, the validation set RV^2^ achieved 0.9412, the RMSEV was 17.0324 mg kg^−1^, and the prediction set was RP^2^ = 0.9648, RMSEP = 12.2429 mg kg^−1^ and UVE-GBM: for the calibration set (RC^2^ = 0.9964, RMSEC = 4.3168 mg kg^−1^), the validation set RV^2^ achieved 0.9304, the RMSEV was 18.5337 mg kg^−1^, and the prediction set was RP^2^ = 0.9648, RMSEP = 12.2323 mg kg^−1^. Figure 5 shows a scatter plot of the reference value and prediction value for Cu content using the CARS-GBM model. Although for the full spectra, GBM was the best (Table 2), for the calibration set RC^2^ = 0.9979, RMSEC = 3.2885 mg kg^−1^, the validation set RV^2^ achieved 0.9308, the RMSEV was 18.4815 mg kg^−1^, and the prediction set was RP^2^ = 0.9596, RMSEP = 13.1156 mg kg^−1^. It can be seen in Figure 5a,b that the prediction result from GBM combined with CARS is better than the result from the full wavelength. GBM-CARS is better for Cu because it is able to capture interactions between variables.

#### 3.2.3. Pb Content Prediction Using Full and Selected Variables

**Figure 6:**
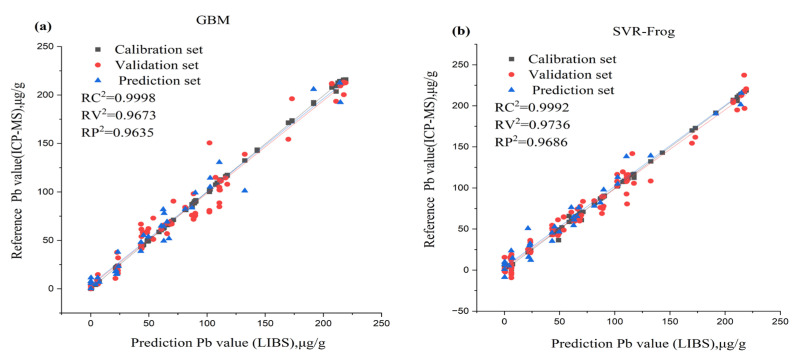
(**a**) Scatter plots of the full variables and reference value (**b**) Scatter plots of SVR combined with RF feature selection method and reference value for the content of Pb.

As shown in Table 3, RF combined with SVR produced the best result for the variable selection methods for Pb content prediction with the following calibration set: RC^2^ = 0.9992, RMSEC = 1.6707 mg kg^−1^; the validation set RV^2^ achieved 0.9736, the RMSEV was 10.2323 mg kg^−1^, and the prediction set was RP^2^ = 0.9686, RMSEP = 10.1224 mg kg^−1^. This was followed by UVE-GBM: calibration set RC^2^ = 0.9992, RMSEC = 1.7248 mg kg^−1^, the validation set RV^2^ achieved 0.9562, the RMSEV was 13.1967 mg kg^−1^, and the prediction set was RP^2^ = 0.9609, RMSEP = 11.3113 mg kg^−1^ and CARS-PLSR: calibration set RC^2^ = 0.9683, RMSEC = 11.2039 mg kg^−1^, the validation set RV^2^ achieved 0.9718, the RMSEV was 10.5798 mgkg^−1^, and the prediction set was RP^2^ = 0.9599, RMSEP = 11.4563 mg kg^−1^. Although for the full spectra, GBM was also the best (Table 2), for the calibration set RC^2^ = 0.9998, RMSEC = 0.8488 mg kg^−1^, the validation set RV^2^ achieved 0.9673, the RMSEV was 11.3933 mg kg^−1^, and the prediction set was RP^2^ = 0.9635, RMSEP = 10.9220 mg kg^−1^. It can be noticed that the prediction result for SVR combined with RF, not GBM, as it is with Cd and Cu, as shown in the Figure 6a,b, it is slightly better than that for the full wavelength. SVR-RF is better for Pb because it is able to capture non-linear relationships between variables.

## 4. Discussion

Among the perennial herbaceous plants found mainly in the Zhejiang, Jiangsu, and Anhui provinces of China, *Fritillaria thunbergii* Miq. [11] was selected as the research object for collecting LIBS data and assessing its heavy metal contents (Cd, Cu, and Pb). The presence of heavy metals in excess of the standard poses a significant health risk. Due to increasing regulation of (TCM), heavy metals have become a priority pollutant in TCM and a serious safety concern [14,15,16]. Thunbergii fritillaria bulbs (Zhebeimu) are most commonly used in Chinese medical clinical practice to treat coughs caused by wind-heat and phlegm-heat, bronchitis, inflammation, hypertension, gastric ulcers, diarrhea, and bacterial infections [12].

In addition, Zhebeimu is widely used in the treatment of leukemia that is resistant to drugs [13]. Despite the development of fast and clean methods for TCM analysis [47]. Using LIBS to detect heavy metals in Fritillaria Thunbergii has still not been reported. Therefore, the ability to detect heavy metals in different varieties of Fritillaria using LIBS technology is a unique and vital process for establishing effective environmental control strategies and monitoring human exposure to heavy metals. In light of the above considerations and the advantages of the LIBS technique, in this study, a quantitative and simultaneous analysis of the contents of three heavy metals in *Fritillaria thunbergii* was performed. LIBS data, however, typically show a high degree of covariance due to the ability of LIBS to measure multiple emission lines from the same species or element; besides, several variables are responsible for creating each peak. Therefore, selecting variables may result in a reduction in the covariance and complexity of the model. It is also helpful to select more explanatory variables because this allows a better understanding of the multivariate system in terms of its chemical characteristics [56]. As well as improving the model fit and increasing the explained variance, removing noisy areas, such as extreme regions of the spectrometer and x variables with no analytical information, can also help to remove noisy regions [56].

In multivariate analysis, the matrix effect and fluctuations in the LIBS spectrum can be taken into account, in addition to the fluctuation in the LIBS spectrum from shot to shot. Recent years have seen an extensive use of chemometric methods, such as partial least squares (PLSR) and support vector machines (SVM) in the analysis of LIBS spectra for multivariable analysis [66,67]. An analysis of three variable selection methods (CARS, RF, and UVE) was conducted in order to simplify the calculation process and improve model performance. PLSR, SVM, and GBM models were run on the full spectra and spectral variables from CARS, RF, and UVE, respectively. A comparison of the performance of PLSR, SVM and GBM models was made by determining the root mean square error of calibration and prediction (RMSEC and RMSEP), as well as the greatest correlation coefficient square (R^2^) between calibration and prediction sets (RC^2^ and RP^2^). It has been found that the combination of SVR and CARS yielded the smallest number of variables selected.

**Figure 7:**
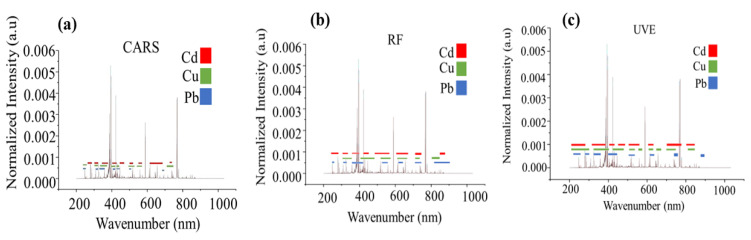
(**a**) Distributions of selected wave number by CARS (**b**) Distributions of selected wave number by RF (**c**) Distributions of selected wave number by UVE.

The combination of GBM with UVE yielded the greatest number of variables for Cd and Cu, and for Pb, GBM with RF yielded a limited number of variables (Table 3). The results of the different feature selection methods differ when compared when calibration, validation, and prediction sets are analyzed. Different feature selection methods were best for the different heavy metals. For Cd: GBM combined with UVE obtained the best performance; for Cu: GBM combined with CARS obtained the best performance, whereas for Pb: SVR combined with RF obtained the best performance. Several studies have demonstrated that there is no single technique for selecting features that is universally optimal [68], and multiple subsets of features are usually equally effective in predicting the data [69,70,71]. There is no doubt that full spectra contain essential information for elemental analysis but inevitably contain irrelevant information and noise, which weaken the model’s capability [63,72,73]. The data analysis step in LIBS, as in other fields of spectroscopic analysis, is heavily constrained both by the high-dimensional input spaces and their inherent sparsity. In addition to reducing the measurement and storage requirements for LIBS data, properly selecting spectral features can facilitate the visualization and understanding of the data and enable more timely and cost-effective classification methods to be developed. The selection of feature variables needs to be applied to reduce computational complexity. As described above, it is worth noting that the number of variables selected by different feature selection methods varies widely (Table 3). For Cd, CARS showed the least variables (37), followed by RF (151) and UVE (192) and for Cu, CARS also showed the least variables (66), followed by RF (120), UVE (231) and finally for Pb, CARS showed the least variables (17), followed by UVE (93), RF (124), as can be seen from Figure 7. All methods select the informative region around 200–1000 nm, which is consistent with the Cd, Cu and Pb results in previous literature [1,37,39,40,48,63,74,75], indicating that these variable intervals are the informative variables.

The different feature selection algorithms indicate some essential variables, so the variables in those intervals must be supported to improve the model’s prediction ability, indicating those regions are informative variable intervals. Table 3 summarizes the results of CARS, RF, and UVE variable selection methods. Based on Figure 5, all selection methods perform significantly better in the test set than when compared with the full spectrum. The spectral matrix was then transformed by reducing the spectral information of the LIBS measurements to the most relevant variables that contained the most relevant spectral information of the respective heavy metals in Fritillaria. Multivariate analysis could address laser-to-sample interaction, experimental parameter variance, and matrices, among other factors [76].

## 5. Conclusions

The study indicated that when using the wavelength selection method, only a limited number of useful variables were extracted, and non-informative variables were eliminated. Therefore, the study explored effective variables by variable selection methods. The experimental results showed that all three methods applied could accomplish variable selection effectively, among which GBM-UVE for Cd, SVR-RF for Pb, and GBM-CARS for Cu produced the best results. Results of some recent heavy metal detection using LIBS are presented in Table 4 for comparison with the current work. Table 4 provides a comprehensive comparison of the results from the current work with those obtained from recent LIBS studies. It allows for a direct comparison of the accuracy of the detection methods. This study demonstrated the potential of LIBS coupled with variable selection and chemometrics as a tool for the rapid detection of heavy metals in varieties of *Fritillaria thunbergii*. It is essential to select variables that are associated with spectral information, so that subsequent modeling analysis can be based on more concise and effective spectral data. Additionally, the wavelengths selected could provide a theoretical foundation for the development of new instruments.

## Figures and Tables

**Table 1:** Heavy metals (Cu, Cd and Pb) contents of Fritillaria obtained by ICP-MS.

Heavy Metal	Groups	CK	1	2	3	4	5	6	7
	Number	36	36	36	36	36	36	36	36
Cu	Min.	1.50	13.80	33.01	42.51	53.76	66.49	155.41	169.66
	Max.	4.88	27.99	55.97	61.28	69.33	82.97	242.04	245.36
	Mean	2.50	20.06	37.31	52.48	65.95	79.66	172.57	215.06
	Range	3.37	14.18	22.95	18.76	15.57	16.47	86.63	75.69
	Var.	1.20	13.14	36.71	37.67	16.87	20.33	517.21	773.97
	Std	1.09	3.62	6.05	6.13	4.10	4.50	22.74	27.82
Cd	Min.	0.17	5.21	9.64	20.81	24.73	44.85	63.06	83.66
	Max.	1.19	7.08	11.70	25.34	29.38	97.26	117.89	100.65
	Mean	0.43	5.86	10.64	22.87	26.39	60.32	82.48	93.91
	Range	1.02	1.87	2.05	4.52	4.65	52.41	54.82	16.98
	Var.	0.07	0.21	0.31	2.34	1.72	394.02	175.37	35.38
	Std.	0.28	0.46	0.56	1.53	1.31	19.85	13.24	5.94
Pb	Min.	0.13	4.12	21.28	43.13	58.61	63.14	102.16	143.12
	Max.	0.76	7.33	23.90	102.03	71.15	90.51	112.26	219.07
	Mean	0.29	6.06	22.75	51.58	65.62	85.00	112.26	199.24
	Range	0.63	3.21	2.61	58.90	12.53	27.36	30.33	75.95
	Var.	0.03	0.56	0.64	265.51	14.55	59.54	63.84	604.43
	Std.	0.19	0.74	0.80	16.29	3.81	7.71	7.99	24.58

CK: Control group.

**Table 2:** Prediction results of the different models using full variables.

	Models	Variables	RC^2^	RMSEC	RV^2^	RMSEV	RP^2^	RMSEP
Cd	SVR	Full variables	0.9999	0.0995	0.8957	11.0874	0.8276	14.5054
	PLSR	Full variables	0.9923	3.0637	0.6791	19.4539	0.5499	23.4405
	GBM	Full variables	0.9984	1.3693	0.8924	11.2625	0.9139	10.2494
Cu	SVR	Full variables	0.9999	0.0995	0.9606	13.9359	0.9280	17.5096
	PLSR	Full variables	0.9936	5.7691	0.6902	39.1138	0.5622	43.1827
	GBM	Full variables	0.9979	3.2885	0.9308	18.4815	0.9596	13.1156
Pb	SVR	Full variables	0.9999	0.0996	0.9304	16.6308	0.8876	19.1794
	PLSR	Full variables	0.9938	4.9443	0.6925	34.9791	0.6272	34.9311
	GBM	Full variables	0.9998	0.8488	0.9673	11.3933	0.9635	10.9220

RC^2^: Coefficient of determination for calibration; RV^2^: Coefficient of determination for validation; RP^2^: Coefficient of determination for prediction; RMSEC: Root mean square error for calibration; RMSEV: Root mean square error for validation; RMSEP: Root mean square error for prediction.

**Table 3:** Prediction results of the different models using three variable selection (CARS, RF and UVE) methods.

	Elements	Methods	N	RC^2^	RMSEC	RV^2^	RMSEV	RP^2^	RMSEP
SVR	Cd	CARS	37	0.9707	5.9935	0.9235	9.4984	0.8139	15.0703
		RF	151	0.9999	0.1000	0.9287	9.1671	0.9322	9.0933
		UVE	192	0.9525	7.6277	0.9066	10.4929	0.9116	10.3842
	Cu	CARS	66	0.9803	10.1823	0.9622	13.6476	0.9430	15.5721
		RF	120	0.9860	8.5699	0.9640	13.3319	0.9658	12.0658
		UVE	231	0.9715	12.2395	0.9412	17.0324	0.9648	12.2429
	Pb	CARS	17	0.9869	7.1947	0.9710	10.7251	0.9341	14.6841
		RF	124	0.9992	1.6707	0.9736	10.2323	0.9686	10.1224
		UVE	93	0.9799	8.9112	0.9585	12.8355	0.9395	14.0633
PLSR	Cd	CARS	37	0.9315	9.1659	0.9098	10.3132	0.9060	10.7075
		RF	151	0.9825	4.6313	0.9313	9.0014	0.9182	9.9903
		UVE	192	0.9659	6.4658	0.8910	11.3386	0.9072	10.6405
	Cu	CARS	66	0.9758	11.2746	0.9520	15.3841	0.9568	13.5625
		RF	120	0.9769	11.0291	0.9385	17.4200	0.9457	15.1970
		UVE	231	0.9856	8.7040	0.9411	17.0462	0.9575	13.4396
	Pb	CARS	17	0.9683	11.2039	0.9718	10.5798	0.9599	11.4563
		RF	124	0.9844	7.8436	0.9303	16.6519	0.9129	16.8794
		UVE	93	0.9720	10.5175	0.9556	13.2832	0.9381	14.2331
GBM	Cd	CARS	37	0.9907	3.3649	0.9009	10.8113	0.9146	10.2061
		RF	151	0.9982	1.4753	0.8474	13.4134	0.8909	11.5370
		UVE	192	0.9967	1.9853	0.8899	11.3934	0.9403	8.5344
	Cu	CARS	66	0.9933	5.9332	0.9316	18.3779	0.9665	11.9356
		RF	120	0.9929	6.0952	0.9371	17.6235	0.9545	13.9099
		UVE	231	0.9964	4.3168	0.9304	18.5337	0.9648	12.2323
	Pb	CARS	17	0.9970	3.4434	0.9469	14.5335	0.9429	13.6631
		RF	124	0.9982	2.6103	0.9329	16.3340	0.9136	16.8175
		UVE	93	0.9992	1.7248	0.9562	13.1967	0.9609	11.3113

N: Number of features selected; RC^2^: Coefficient of determination for calibration; RV^2^: Coefficient of determination for validation; RP^2^: Coefficient of determination for prediction; RMSEC: Root mean square error for calibration; RMSEV: Root mean square error for validation; RMSEP: Root mean square error for prediction.

**Table 4:** Results of some recent heavy metals (Pb, Cd and Cu) detection using LIBS of various samples and current work.

Heavy-Metal	Sample	Spectral Line (nm)	Reference
Cd	Lettuce	214.44, 226.50, 228.80	[63]
	Sargassum fusiforme	441.56, 643.85	[37]
	Lipstick	467.9, 573.80	[23]
	This work	214.44, 226.50, 441.56, 467.90, 573.80, 643.85	
Cu	Traditional Chinese medicinal materials	324.79, 327.35	[74]
	Glycyrrhiza	324.70	[75]
	Ligusticum wallichii	324.46, 327.09	[40]
	Sargassum fusiforme	324.75, 327.40	[37]
	Rice	324.754, 327.396	[48]
	This work	324.09, 324.79	
Pb	Paint samples	405.70	[77]
	Henna paste	405.78	[39]
	Ligusticum wallichii	405.80	[40]
	Medicinal herbs	405.78, 404.00	[1]
	This work	280.00, 404.00, 405.70	

## Data Availability

The data presented in this study are available on request from the corresponding author.
